# When beats are hard to crack: electrical storm in a patient with severe myocardial calcification

**DOI:** 10.1093/ehjcr/ytaf197

**Published:** 2025-05-14

**Authors:** Edgar Francisco Carrizales-Sepúlveda, Carlos E Guzmán, Timothy C Tan

**Affiliations:** Heart Failure Unit, Cardiology Service, Hospital Universitario ‘Dr. José E. González’, Universidad Autónoma de Nuevo León, Madero and Gonzalitos Av N/N, Mitras Centro, Monterrey, Nuevo León 64460, México; Electrophisiology Laboratory, Cardiology Service, Hospital Universitario ‘Dr. José E. González’, Universidad Autónoma de Nuevo León, Madero and Gonzalitos Av N/N, Mitras Centro, Monterrey, Nuevo León 64460, México; Cardiology Department, Hospital Christus Muguerza de Alta Especialidad, Miguel Hidalgo y Costilla Av 2525, Obispado, Monterrey, Nuevo León 64060, México; Department of Cardiology, Blacktown Hospital, University of Western Sydney, Blacktown Road, Blacktown, NSW 2148, Australia; School of Medical Sciences, Faculty of Medicine, University of New South Wales, Kensington, NSW 2052, Australia

**This editorial refers to ‘The Role of Multimodal Cardiac Imaging in Managing Electrical Storms in Severe Heart Calcifications - a Case Report’, by A. Lejeune *et al*. https://doi.org/10.1093/ehjcr/ytaf068.**

Extensive myocardial calcification (MC) is a rare and severe form of myocardial disease associated with an adverse prognosis. However, recommendations on how to better diagnose, treat, and monitor this disease entity are lacking, representing a real clinical challenge.^1–3^ Myocardial calcifications are broadly categorized into dystrophic or metastatic (*Figure 1*).^1^ Dystrophic calcifications, which are the most common type, are secondary to calcium deposition in previously injured myocardium. Myocardial infarction is usually described as the most common cause, but some recent reports suggest that sepsis and myocarditis represent the most common causes in the late years, as contemporary reperfusion strategies limit myocardial damage, avoiding this complication.^3^ Metastatic calcifications are less common representing ∼ 20% of the cases, and associated with systemic processes related to alterations in calcium homoeostasis with chronic kidney disease among the most common causes. In this type of MC, calcium can deposit in healthy or diseased myocardium and localization of calcifications can be more diffuse.^1,3^

**Figure 1:**
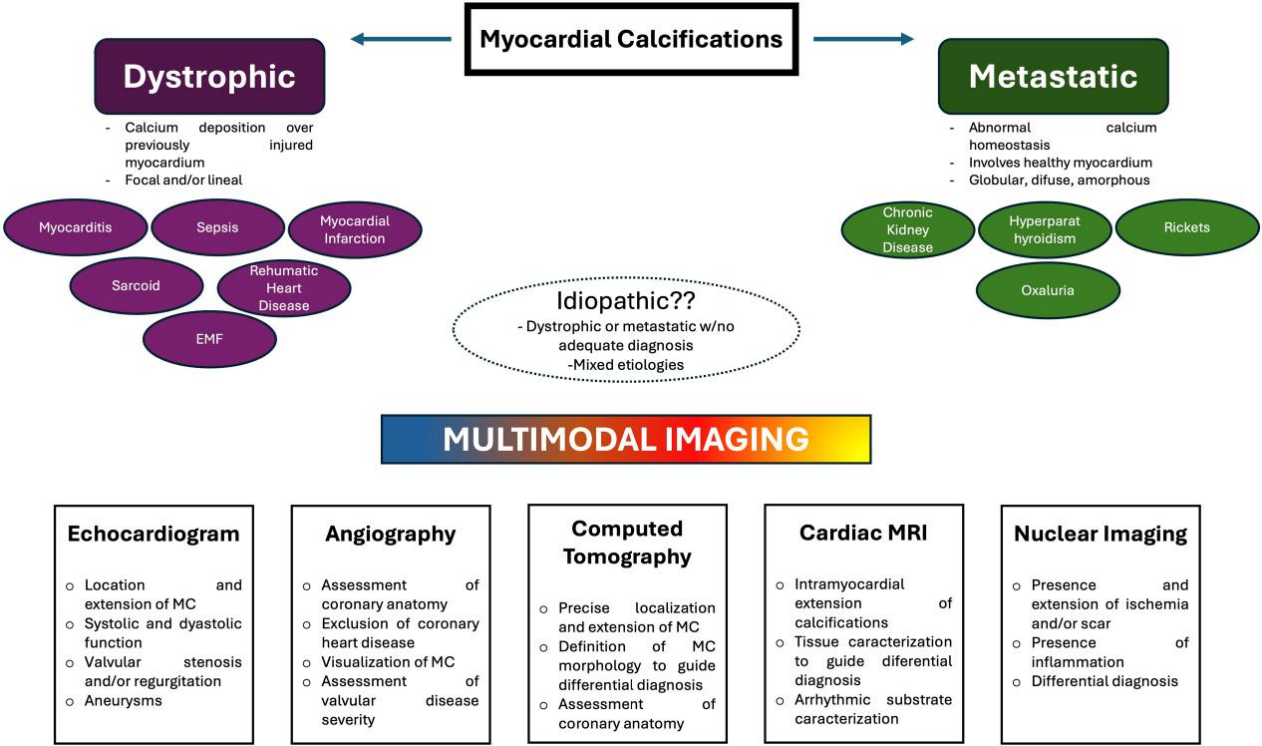
Differential diagnosis of myocardial calcifications and the role of multimodal imaging.

## Myocardial calcifications: a diagnosis and treatment challenge and the value of multimodal imaging

In this issue of the journal, Lejeune *et al.*,^4^ present a complex case of MC complicated by recurrent arrhythmias. The featured patient was diagnosed with MC at the age of 5 years with the first onset of supraventricular arrhythmia at the age of 20 and the first episode of ventricular arrhythmia at the age of 24 years. A transthoracic echocardiogram performed following the initial episode of ventricular tachycardia (VT) demonstrated preserved ventricular function with extensive calcification of the left atrium, mitral valve, and annulus. An extensive diagnostic work-up did not identify a clear aetiology for the MC. The patient underwent an endo-epicardial ablation for management of the VT but had recurrence of VT post-procedure, hence had an implantable cardioverter defibrillator (ICD) placed, he received sotalol later switched to amiodarone but discontinued due to hepatitis and finally placed on bisoprolol. Interestingly, aside from the MC, the patient’s medical history was also significant for two transient ischaemic attacks (one at the age 20 years when he had presented with the supraventricular arrhythmia and the second one at the age of 38 years), an acute coronary event at the age of 37 years diagnosed as a non-ST-segment elevation myocardial infarction (NSTEMI) which was managed with medical therapy, and severe mitral regurgitation secondary to mitral valve prolapse with associated left ventricular (LV) dilation and pulmonary hypertension which was managed surgically with a mitral valvuloplasty at the age of 40 years. The patient then presented with a VT storm lasting for 4 h with repeated attempts by the ICD to terminate the tachycardia including anti-tachycardic pacing and five shocks, without response. The transthoracic echocardiogram at the time he had presented with the VT storm demonstrated evidence of extension of the previous known calcifications into the aorto-mitral curtain and the inferobasal, posterobasal, and laterobasal LV walls, but overall preserved LV function. Computed tomography (CT) imaging confirmed the presence of large amorphous calcifications within the left atrium walls, mitral valve and annulus, and the LV.^4^

The differentials for MC is broad hence the identification of the aetiology and management can be challenging. Echocardiography, which is usually the first line imaging modality, can reveal evidence of myocardial calcifications, typically as echo densities with posterior acoustic shadowing, and enable identification of associated regional wall motion abnormalities and LV dysfunction, valvular dysfunction secondary the calcification i.e. stenosis or regurgitation and the presence of associated cardiac structural abnormalities i.e. ventricular aneurysms that could suggest prior myocardial infarctions.^1,3,5^ Computed tomography imaging is also extremely helpful in identifying the precise location and extent of the calcifications; with the morphology of the calcified regions frequently providing valuable clues to the potential aetiology.^1^ Cardiac magnetic resonance (CMR) imaging can be used to delineate intramyocardial extension of the calcifications, and to identify the presence of late gadolinium enhancement (LGE) in the surrounding tissue and to differentiate between a possible damage due to previous ischaemia vs. a primary cardiomyopathy associated with myocardial calcifications, such as endomyocardial fibrosis.^6,7^

In the current case, despite the use of multimodal imaging, the precise aetiology of MC could not be clearly defined.^4^ Common systemic causes were excluded, and despite a remote history of a previous myocardial infarction, imaging excluded the presence of ischaemic scars. The calcifications in this case appeared to be focal and present even in early childhood, suggesting a possible early insult, such as rheumatic fever or myocarditis, since these have previously been reported as potential causes during childhood. There have also been rare reports suggesting that even events during the peripartum period could cause MC.^8^ There was evidence of an interval increase in the calcifications, with extension of the areas of calcification demonstrated with a series of complications which could all have potentially arisen from the MC. When a clear aetiology cannot be identified calcifications are usually classified as idiopathic, but in many cases these represent dystrophic, metastatic or mixed causes of calcifications as could have been the case in this patient.^1^ Other imaging techniques like cardiac FDG-PET may have helped to assess for other causes of calcifications like sarcoid, which has also been associated with ischaemic attacks in the young.^9,10^

The patient had an electrical storm (ES) which makes this case unique since ES in the context of MC has not been previously described.^3^ Electrical storms typically require both a substratum and a trigger.^11^ The electrocardiograms suggested that the VT source was from at least two different sites close to the calcified areas and were independent and remote circuits. Aside from the diagnostic work-up, multimodality imaging can also have a role in the management of complex arrhythmias, LGE-CMR and CT with perfusion techniques help in the characterization of myocardial tissue and arrhythmic substrate, and its combination with electrophysiological mapping forms the basis of complex ablation procedures.^12^ Ablation in the context of such severe structural abnormalities (MC) implies endo-epicardial approaches with extensive ablation lesions that can cause collateral myocardial damage in an already damaged myocardium which has been associated with a potential proarrhythmic effect.^13^ In addition, repeated ICD therapies can cause myocardial injury with catecholamine release, sympathetic overactivity inducing automaticity, and/or delayed afterpotentials causing a greater dispersion in ventricular refractoriness which can trigger or perpetuate arrhythmias.^11^ Arrhythmic substrate in this patient was probably a combination of extensive MC and myocardial injury associated with past insults, likely including previous failed ablations and ICD shocks, sympathetic overactivation probably played a role in the perpetuation of the arrhythmias, this could explain the response to sedation and beta-blockers in this patient. Cardiac autonomic imbalance destabilizes normal electrophysiology and promotes ventricular arrhythmogenesis, in these severe and complex scenarios, neuromodulatory therapies aiming to correct sympatho-vagal imbalance show good results.^11,13^

There are some limitations that should be considered when using imaging to evaluate MC, the most relevant being the fact that extensive calcifications can produce artefacts that could limit the evaluation of the disease.^1,3^ There is no specific treatment for extensive MC; limiting the damage associated with the primary trigger could be a valuable way to prevent the development of calcifications, and this is supported by the fact that the incidence of MC secondary to coronary syndromes has been reduced due to contemporary reperfusion strategies.^3^ In this same way, an adequate and timely management of sepsis and the use of cardioprotective medications in patients with myocarditis could help to reduce the risk of developing MC. Treatment of MC mainly involves treating its complications, using diuretics and guideline-directed medical therapies for patients that develop heart failure and, like the present case shows, treating life-threatening arrhythmias.

This patient had a complex case of MC and highlighted some of the challenges typically encountered, and the value of multimodality imaging and a multidisciplinary approach in the management of this condition. This case, apart from demonstrating the potential complexity of MC and the management challenges, describes a unique presentation as an ES, which adds to the myriads of clinical scenarios that this entity can present. Sympathetic overactivation acting on a severely diseased myocardium can trigger and perpetuate arrhythmias, and as shown in this case, neuromodulation can help to control the arrhythmias.

## Lead author biography


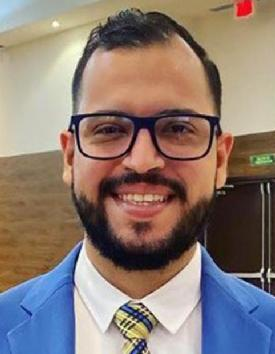


Dr Edgar Francisco Carrizales-Sepúlveda is a professor of cardiology and head of the Heart Failure Clinic at the Cardiology Service, Hospital Universitario, UANL in Monterrey, México. He is also an associated editor of the *European Heart Journal – Case Reports*. His areas of interest are heart failure and myocardial disease.

**Funding:** None.

## Data Availability

The data underlying this article will be shared on reasonable request to the corresponding author.
